# Lower limb blood flow responses during higher‐ and lower‐load bilateral leg press exercise

**DOI:** 10.1113/EP092888

**Published:** 2026-08-11

**Authors:** Eudoxia Zafiris, Jeremy. N. Cohen, Tania J. Pereira, Jason S. Au

**Affiliations:** ^1^ Department of Kinesiology and Health Sciences University of Waterloo Waterloo Canada

**Keywords:** probe holder, resistance exercise, shear rate, ultrasound, vascular physiology

## Abstract

The local blood flow response to resistance exercise (RE) is complicated by local muscular contractions and large swings in perfusion pressure. The magnitude of these responses might vary according to exercise intensity, number of sets and number of repetitions, and as a result, the local shear environment during RE remains uncharacterized. We examined the effect of RE on superficial femoral artery (SFA) blood flow and shear rate during bilateral leg press in healthy males and females. Twenty‐four healthy adults (24 ± 3 years; 14 female) performed either higher‐load lower‐repetition (75% 1‐repetition maximum (RM); 8 reps/set for 3 sets) or lower‐load higher repetition (35% 1‐RM; 20 reps/set for 3 sets) incline leg press to failure in a random counterbalanced order. The SFA was imaged via duplex ultrasound, and the probe was secured on the right thigh using a limb‐affixed probe holder for continuous imaging of blood velocity. Arterial diameter could not be reliably measured during exercise; exercising blood flow and shear rate were estimated from arterial diameters calculated 10 s post‐exercise. Regardless of loading protocol, RE was characterized by increases in mean blood velocity, blood flow, anterograde shear and retrograde shear rate during exercise (time effects *P* < 0.01; comparisons *P* < 0.05), followed by reactive hyperaemia during rest periods with elevated mean shear and an abolished retrograde shear response. Arterial diameter increased only during rest periods between sets, consistent with the hyperaemic stimulus (*P* < 0.01). This research directly addresses our current knowledge gap regarding the vascular response to RE across different intensities of large muscle group exercise.

## Abstract (highlights)

- **What is the central question of this study?**
  How does leg press exercise impact local blood flow of the lower limbs?
- **What is the main finding and its importance?**
  Using hands‐free ultrasound, continuous blood velocity recordings during leg press exercise revealed oscillatory flow during contractions followed by reactive hyperaemia during rest, regardless of load. Blood velocity remained predominantly anterograde with modest increases in retrograde blood flow despite rhythmic contractions. Overall, the vascular environment during dynamic resistance exercise resembled previous work in aerobic exercise, punctuated by intermittent hyperaemia, a shear pattern that may help explain vascular changes with chronic resistance training.

## 1 INTRODUCTION

Exercise training provides transient challenges to the vascular environment, thereby improving chronic cardiac and vascular function, and ultimately reducing future cardiovascular disease risk (Green et al., 2017; Lopes et al., 2021; Pinckard et al., 2019; Whelton et al., 2002). Resistance exercise (RE) has recently gained attention as an effective modality for improving cardiovascular health, with the American Heart Association supporting the role of RE as a supplement to aerobic exercise in a wide range of individuals (Paluch et al., 2024). Physiologically, RE stimulates transient increases in blood pressure, blood flow and shear rate in the active limb, driven by local vasodilation in response to increased metabolic demands (Laughlin et al., 2008; Saltin et al., 1998; Stebbings et al., 2013; Thomas et al., 2020). The increase in blood flow generates higher arterial wall shear stress, though the exact stimulus is complicated by local muscular contractions and large swings in perfusion pressure that interrupt the typical multi‐phasic blood velocity pattern (Green et al., 2017). The prevailing theory is that this shear stress stimulation contributes, in part, to training‐related improvements in vascular function over time, and that factors such as intensity, number of sets, repetitions and modality will dictate the specific shear environment (Birk et al., 2012; Thijssen et al., 2010; Thijssen, Dawson, Black et al., 2009, Thijssen, Dawson, Tinken et al., 2009; Thomas et al., 2020). Even though the active shear environment appears critical to understand vascular changes, a large amount of previous work has focused on the systemic vascular responses in non‐active limbs or post‐exercise responses in active limbs during aerobic exercise; therefore, there is a notable need to characterize the local shear environment of the active limbs during RE (Thomas et al., 2020; Westcott, 2012).

Doppler ultrasound is a useful tool used to determine blood velocity in vessels and measure dynamic changes in vessel diameter; however, due to challenges with continuous imaging during exercise, the haemodynamic environment during bouts of RE is largely understudied. Measuring the local shear environment during exercise has emerged as one of the key challenges in modern cardiovascular physiology (Notarius et al., 2018). We have previously demonstrated feasibility and reliability of a technique for hands‐free continuous blood flow measurements using a limb‐affixed probe holder during lower limb cycling, providing recordings in both active and inactive limbs during aerobic exercise (Cohen et al., 2025). The use of hands‐free probe holders provides considerable promise for high‐throughput data collection in vascular exercise studies, including benefits such as stabilizing the probe relative to the limb, minimizing movement and operator artifact, and alleviating the training time and injury burden on sonographers when performing highly specialized imaging tasks (Evans et al., 2009). Furthermore, while previous manual imaging studies have largely focused on the post‐exercise hyperaemic response to RE, probe holders allow haemodynamic assessment during exercise, when the mechanical stimulus is active.

The purpose of this study is to examine the effect of RE on superficial femoral artery (SFA) blood flow and shear rate during bilateral leg press in healthy adults. Given that RE loads can vary widely, we aimed to investigate how different loading protocols (higher‐load lower‐repetition vs. lower‐load higher‐repetition) influence haemodynamic load. We hypothesized that SFA blood flow would transiently develop highly pulsatile patterns consistent with large swings in transmural pressure induced by local muscle contractions (Thijssen, Dawson, Black et al., 2009). Additionally, we expected the post‐exercise response to be hyperaemic, with an increase in blood flow following the cessation of resistance efforts.

## 2 METHODS

### 2.1 Ethical approval

The study was approved by the University of Waterloo Office of Research Ethics (ORE# 45619) and complied with the *Declaration of Helsinki* regarding the use of human participants. The study was preregistered on OSF prior to beginning data collection (DOI: 10.17605/OSF.IO/VG82A). Written informed consent was obtained from all participants prior to participation in the study.

### 2.2 Participants

Twenty‐four healthy adults (14 females, 24 ± 3 years) participated in this study. All participants had no history of cardiovascular disease or use of medications that would significantly affect the cardiovascular system. Prior to arrival, participants were asked to avoid strenuous exercise and alcohol for 12 h, caffeine for 4 h, and large meals for 3 h before each testing session.

### 2.3 Exercise design

Upon arrival, participants completed a 5‐min dynamic lower‐body warm‐up consisting of lunges and quad stretches. They were then seated in a 45‐degree inclined leg press machine, with their legs elevated and feet positioned shoulder‐width apart on the raised platform. Participants were asked to perform 10 unweighted repetitions to complete the warm‐up and were instructed to maintain proper form by limiting knee flexion to approximately 90 degrees at the deepest point of the leg press. A 10‐repetition maximum (10‐RM) was determined by progressively increasing weight based on perceived effort and participant feedback, and subsequently used to estimate 1‐repetition maximum (1‐RM) using the Brzycki formula (https://alphaprogression.com/en/tools/rm‐calculator). Participants then performed either low‐load high‐repetition (35% 1‐RM for 20 reps) or high‐load low‐repetition (75% 1‐RM for 8 reps) bilateral leg presses for two sets with an additional third set performed until volitional failure, in a randomized, counterbalanced order. Target loads for the leg press (35% and 75% of estimated 1‐RM) were rounded to the nearest available increment of the weight stack (±2.3 kg). Repetitions were performed in sync with a 40 Hz metronome. A 2‐min rest was provided between sets, and a 15‐min rest between intensity trials. During each 2‐min rest, participants were instructed to keep their legs elevated and feet pressed against the raised platform for blood flow measurements. If necessary, a researcher provided support by bracing the participants legs to help alleviate fatigue. A brachial blood pressure cuff was placed on the participants left arm to measure blood pressure in triplicate at the start of each intensity and after each completed set. Continuous heart rate monitoring was conducted using a lead II electrocardiogram.

### 2.4 Ultrasound methodology

**FIGURE 1:**
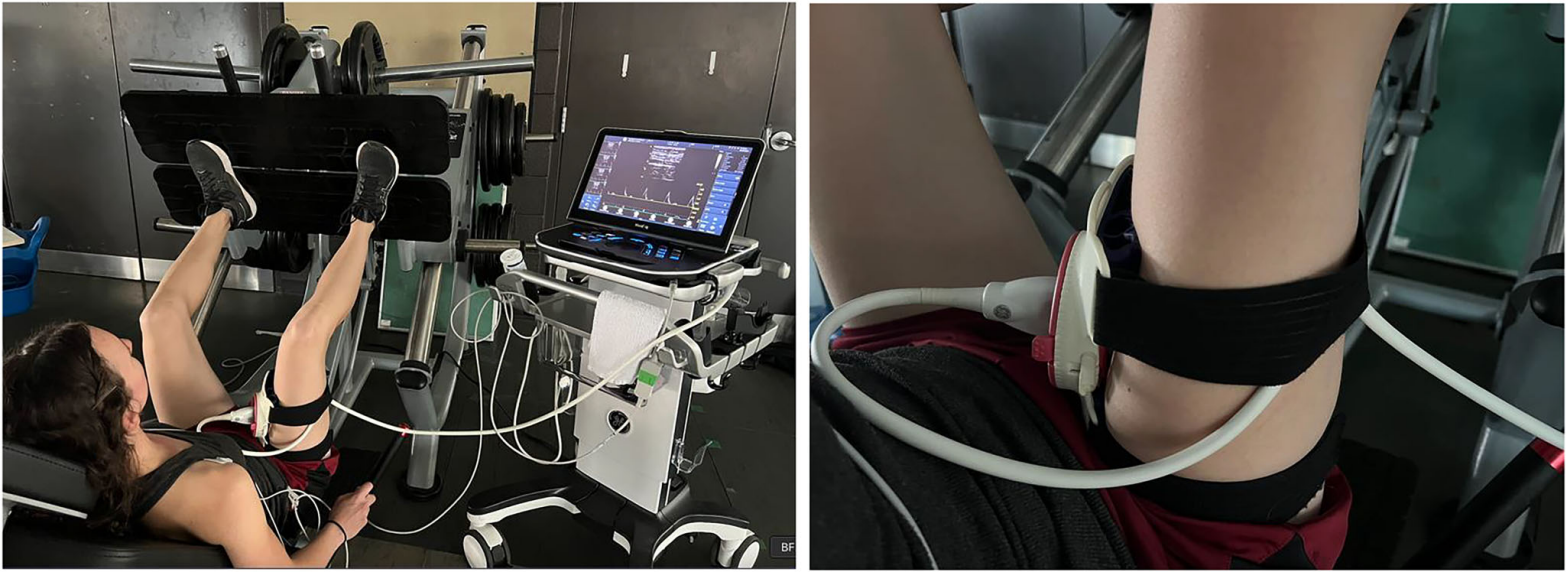
Experimental set‐up showing a participant sitting in the incline leg press with legs elevated and feet pressed against the platform. The probe holder device is affixed to the right thigh, recording continuous duplex blood velocity and arterial diameters from the superficial femoral artery with no manipulation during exercise.

The right SFA was imaged with duplex ultrasound using a 12L linear array probe (Vivid IQ; GE Healthcare, Chicago, IL, USA), which was attached to a probe holder device affixed to the upper thigh (ProbeFix Dynamic (PFD); Usono Inc., Eindhoven, Netherlands) (Figure 1). A trained sonographer located the SFA by scanning the medial aspect of the right mid‐thigh. Once the SFA was identified, the sonographer attached the PFD device to the participant's upper thigh and secured the ultrasound probe at the appropriate imaging site (Cohen et al., 2025). The participant then placed their feet shoulder‐width apart on the raised platform, and minor adjustments were made to the probe positioning to ensure optimal visualization and consistent recording of the SFA. Cine loops were recorded at the SFA for 60 s of baseline data prior to each exercise intensity. Separate cine loop recordings were then captured for each set, covering the entire duration of exercise and the subsequent 2‐min post‐exercise resting period, during which mean blood velocity (MBV) and arterial diameter were captured. The Doppler sampling gate was positioned in the centre of the artery at baseline, with the sample volume expanded beyond the walls of the vessel to anticipate transposition of the artery during muscular contractions. A wall filter was applied to the Doppler settings to reduce artifact associated with tissue motion during muscular contractions. Artery depth was 2–4 cm and B‐mode frame rates ranged from 8–25 fps, depending on the SFA depth. During exercise, participants were instructed to remain mindful of the PFD device and avoid disturbing its location, without compromising exercise form. In cases of misalignment, the sonographer adjusted the PFD device during the rest period to correct the imaging window.

### 2.5 Blood flow analysis

SFA diameter and blood velocity were analysed from the stored cine loops. The diameter was manually measured in triplicate at end‐diastole using the ultrasound's callipers to identify the interface between the intima and lumen, and a mean value was recorded for each measurement. Diameters were obtained for baseline, immediately post‐exercise, and at 1‐ and 2‐min post‐exercise. Due to motion artifact and low B‐mode frame rate in duplex mode, it was not possible to reliably estimate SFA diameter during exercise. Blood velocity was measured using an open‐access MATLAB program (FloWave.US (Coolbaugh et al., 2016)), which calculates MBV based on time‐dependent pixel intensity changes. Blood flow was then calculated as:

$$\mathrm{Blood}\;\mathrm{flow}\left(\mathrm{mL}/\min\right)=\left(\pi \times \mathrm{radiu}s^{2}\right)\times \mathrm{MBV}\times 60\;s/\min$$

**FIGURE 2:**
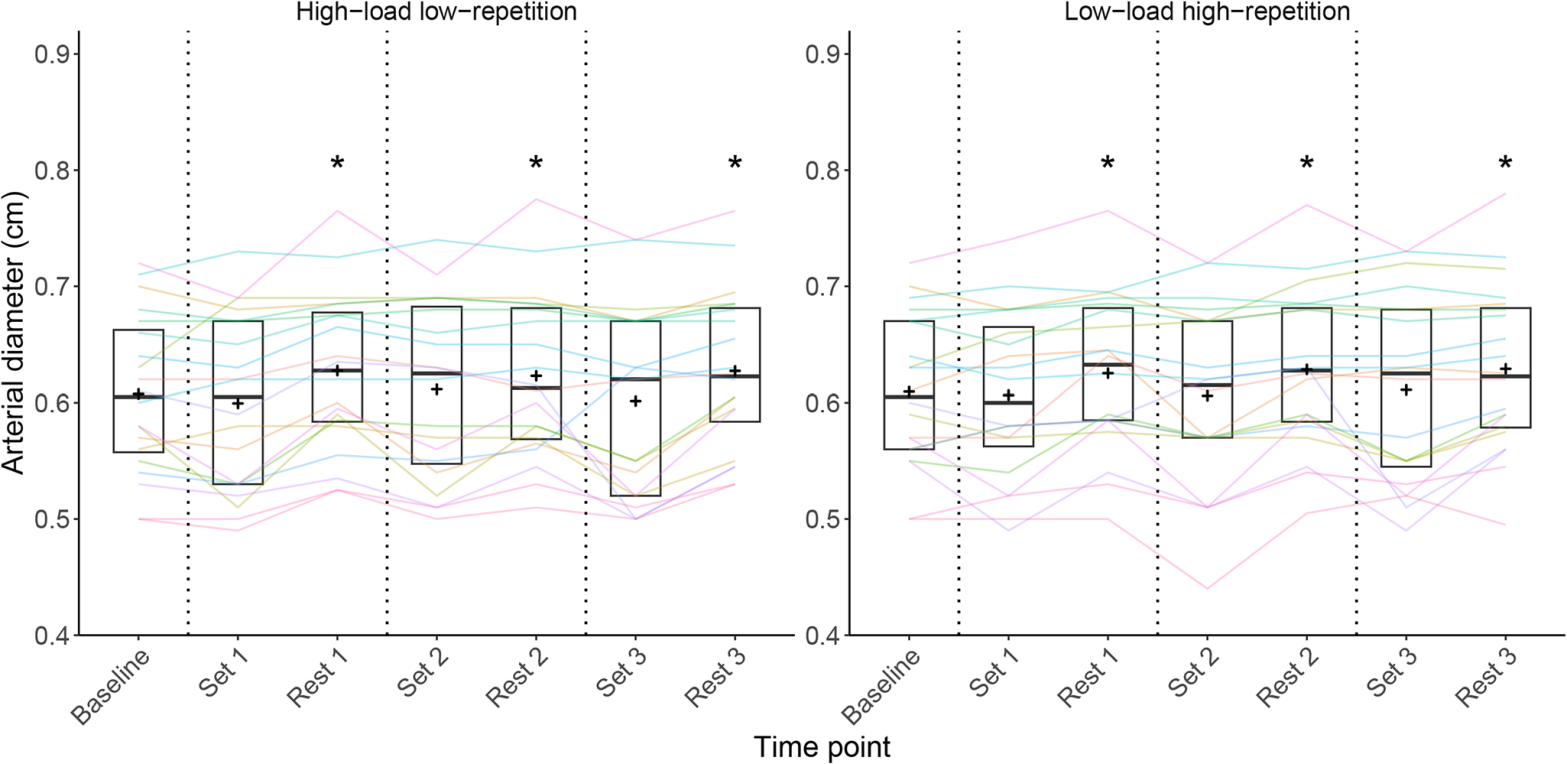
Diameter of the superficial femoral artery during high‐load low‐repetition and low‐load high‐repetition leg press exercise. Measurements in sets 1, 2 and 3 are taken immediately after exercise. Each participant is represented by a coloured line; the boxplots represent the 25th and 75th percentile of the distribution with the median and mean indicated as solid lines and crosses, respectively. *P < 0.05 different from baseline and all exercise sets (main effect of time, *P* < 0.01).

Due to the inability to reliably measure arterial diameter during active contractions, blood flow and shear rate during exercise were estimated with diameter values obtained immediately following exercise cessation for 10 s when the probe holder returned the artery back to a clear window in the absence of muscle contraction. This decision was made during data analysis as clear diameter changes were noted to occur during the hyperaemic rest periods (Figure 2). Therefore, haemodynamic variables reporting ‘during exercise’ should be interpreted with caution and primarily reflect changes in the MBV signal.

### 2.6 Statistics

All quantitative analyses and graphing were performed in R (v4.4) (https://www.r‐project.org/). To determine experimental outcomes, a 7 × 2 repeated‐measures ANOVA was conducted to examine the effects of time (baseline, set 1, rest 1, set 2, rest 2, set 3, rest 3) and condition (high and low intensity) for both blood flow and arterial diameter. Significant main effects were evaluated with *post hoc* pairwise comparisons using a paired Student's *t*‐test with Holm's correction. Additionally, a secondary sex analysis was conducted using a 7 × 2 × 2 repeated‐measures ANOVA to examine the impact of sex (male and female) on changes in blood flow and arterial diameter across trials. Significant main effects were then evaluated with *post hoc* pairwise comparisons using paired and unpaired Student's *t*‐tests with Holm's correction. The type I error rate was set a priori at α = 0.05.

## 3 RESULTS

All participants successfully completed the RE protocol without adverse events (Table 1). Hemodynamic responses to exericse are presented in Table 2. A total of *n* = 4 participants were excluded from the final dataset due to poor ultrasound image quality, including loss of imaging windows during exercise or poor signal quality. A representative example of blood velocity recordings is provided in Supporting information, Video S1. A paired equivalence test (TOST) demonstrated that MBV returned to baseline values after the wash‐out period between intensity trials (equivalence bounds ±5 cm/s; mean difference = 0.56 cm/s, 90% CI: −1.66 to 2.78; *t*(19) = −3.46, *P* = 0.0013).

**TABLE 1:** Participant characteristics.

Variable	Male	Female
*n*	10	14
Age (years)	24 ± 3	24 ± 3
Height (cm)	180 ± 11	172 ± 10
Body mass (kg)	76 ± 9	67 ± 10^*^
10‐RM (kg)	250 ± 49	205 ± 48^*^
1‐RM (kg)	332 ± 65	273 ± 64^*^
35% load (kg)	116 ± 23	96 ± 22^*^
75% load (kg)	250 ± 49	205 ± 48^*^

Data represented as means ± SD.

* *P* < 0.05 vs. Male

**TABLE 2:** Haemodynamic responses immediately following resistance exercise (*n* = 20).

	Baseline	Rest 1	Rest 2	Rest 3
SFA: Low‐load high‐rep				
Systolic blood pressure (mmHg)	127 ± 7	139 ± 11^*^	144 ± 14^*^	147 ± 12^*^
Diastolic blood pressure (mmHg)	76 ± 7	75 ± 6	77 ± 7	77 ± 10
Heart rate (bpm)	82 ± 14	101 ± 14^*^	106 ± 17^*^	120 ± 20^*^
SFA: High‐load low‐rep				
Systolic blood pressure (mmHg)	126 ± 9	141 ± 14^*^	145 ± 15^*^	150 ± 17^*^
Diastolic blood pressure (mmHg)	75 ± 8	74 ± 6	72 ± 6	72 ± 7
Heart rate (bpm)	83 ± 16	105 ± 14^*^	110 ± 18^*^	121 ± 16^*^

*Note*: Data represented as means ± SD.

* *P* < 0.05 vs. Baseline.

### 3.1 Arterial diameter

Arterial diameter could not reliably be assessed during exercise due to considerable motion artifact and the low B‐mode frame rate in duplex mode. Therefore, diameter measurements of the active sets were estimated from the immediate post‐exercise period and used in the calculation of exercise blood flow and shear rates. SFA diameter increased during the resting periods compared to both baseline and immediate post‐exercise measurements (main effect for time *P* < 0.01; comparisons *P* < 0.05; Figure 2).

### 3.2 Blood flow and shear rate

**FIGURE 3:**
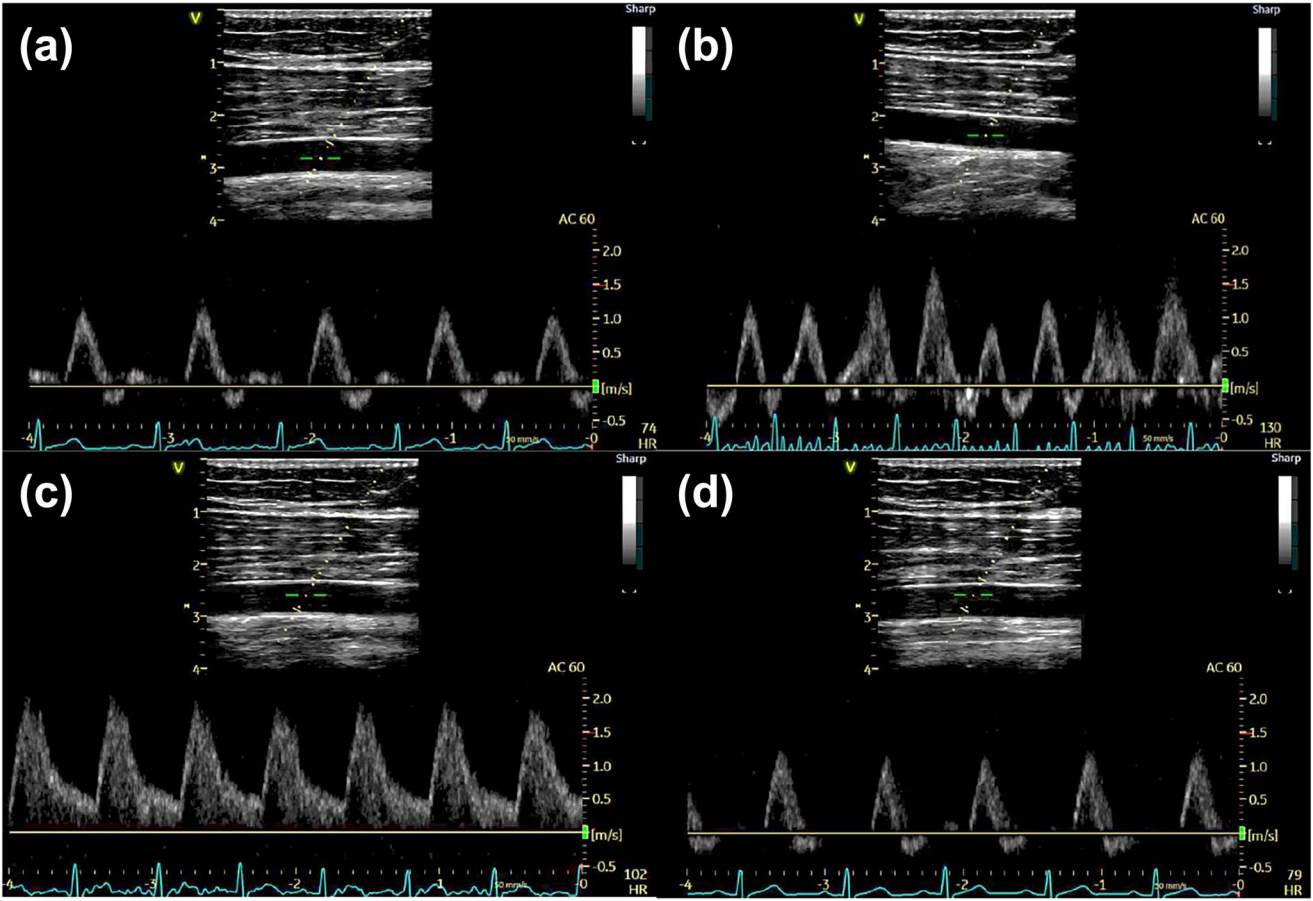
Representative blood flow velocity images obtained from a 21‐year‐old female participant with a hands‐free probe holder during high‐load low‐repetition exercise. Blood velocity and arterial diameters were captured at four time points: (a) baseline, (b) during exercise, (c) after 1 min of rest, and (d) after 2 min of rest. Velocity scales (pulse repetition frequency) are kept consistent throughout the bout of exercise.

**FIGURE 4:**
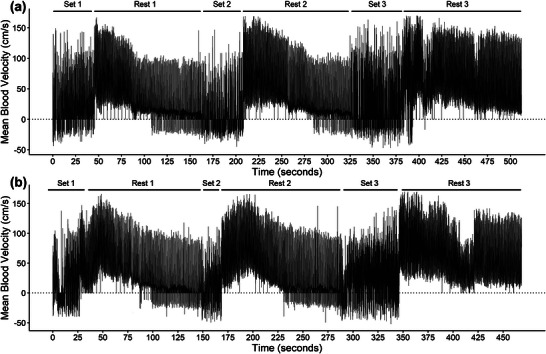
Representative example from a 21‐year‐old female of continuous superficial femoral artery raw mean blood velocity during low‐load high‐repetition (a) and high‐load low‐repetition (b) exercise protocols. The timing of each trial has been indicated by sequentially labelling set and rest periods in each exercise protocol. Each trial begins with exercise (e.g., set 1), followed by a rise in blood velocity immediately after the set is completed (reactive hyperaemia) during the rest period. Each exercise set was followed by a standard 2‐min rest interval, which is consistent across all trials. For presentation purposes, data have only been cleaned for local transient artifacts; no filtered data are presented.

**FIGURE 5:**
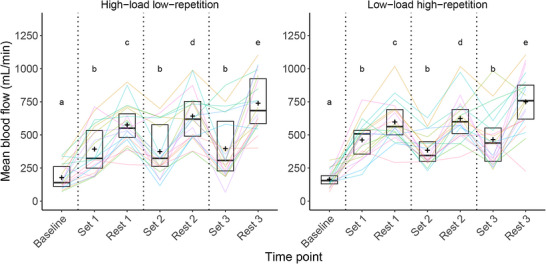
Average blood flow of the superficial femoral artery during high‐load low‐repetition and low‐load high‐repetition leg press exercise. Values during exercise sets are derived from diameter data taken immediately post‐exercise. Each participant is represented by a coloured line; the boxplots represent the 25th and 75th percentile of the distribution with the median and mean indicated as solid lines and crosses, respectively. Different letters indicate significant differences between time points (main effect of time *P* < 0.01; *post hoc* comparisons *P* < 0.05); time points sharing the same letter are not significantly different (i.e., baseline [a] is different from all other time points, whereas there is no difference between any exercise sets [b]).

**FIGURE 6:**
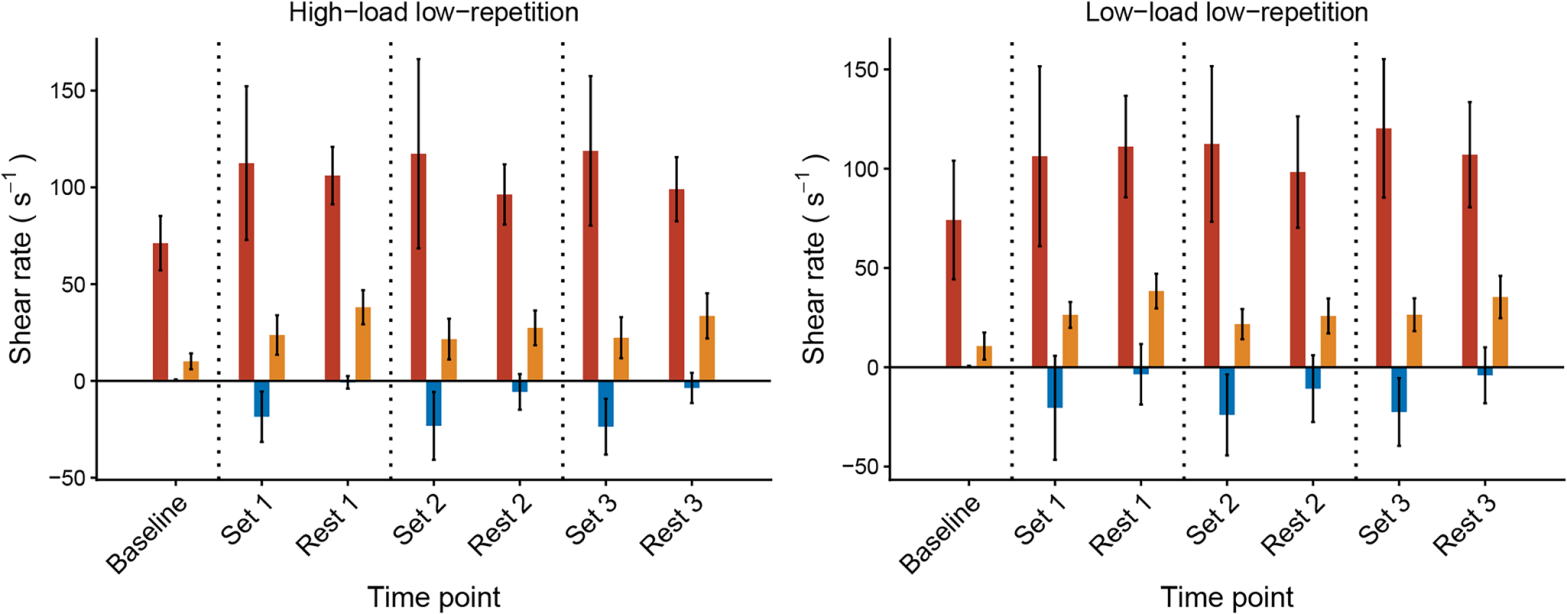
Superficial femoral artery shear rate patterns during high‐load low‐repetition and low‐load high‐repetition leg press exercise. Values during exercise sets are derived from diameter data taken immediately post‐exercise. The shear profile was separated into peak anterograde (red), peak retrograde (blue) and mean shear rate (yellow) at each time point. Statistical significance is omitted from the figure to improve clarity of the visualization. Mean ± SD is indicated by the bars.

Representative images and blood velocity traces are shown in Figure 3 and Figure 4, respectively, demonstrating a continuous recording of blood velocity during rest, exercise, and post‐exercise using a hands‐free probe holder. Blood flow increased during both high‐ and low‐intensity exercise throughout each set and rest period compared to baseline (main effect for time *P* < 0.01; Figure 5). Blood flow progressively increased during the rest periods compared to during exercise in all sets (R3 > R2 > R1; all *P* < 0.05), though there was no increase in exercising blood flow as the sets progressed. Blood flow in each rest period was also elevated compared to all exercise sets (all *P* < 0.05). There was no effect of lifting protocol (main effect for condition *P* = 0.16) nor any interaction between time and condition (interaction effect *P* = 0.35) for mean blood flow. Anterograde shear was increased during all exercise sets and remained elevated during resting periods compared with baseline (all *P* < 0.01; Figure 6). Anterograde shear was slightly reduced during the second and third rest periods compared to their preceding exercise sets (both *P* < 0.01), consistent with the lack of active contractions. Retrograde shear was increased during the exercise periods compared with baseline (all *P* < 0.01), but returned to baseline values during each rest period (*P* > 0.05 not different from baseline, abolishing the oscillatory profile), consistent with reactive hyperaemia in the absence of muscle contraction. Mean shear was increased during all exercise and rest periods compared with baseline (all *P* < 0.01), increasing during rest periods compared with their preceding exercise set (all *P* < 0.05; Figure 6).

### 3.3 Secondary sex analysis

As an exploratory objective, analyses were repeated including sex as another independent variable. There was no interacting effect of sex for mean blood flow (*P* = 0.60) or SFA diameters (*P* = 0.90). Males had larger SFA diameters than females (*P* < 0.01) consistent with known sex differences in artery structure.

## 4 DISCUSSION

This study aimed to describe the impact acute RE has on haemodynamic changes in the SFA during bilateral leg press in males and females. A novel aspect of this study was the use of a hands‐free ultrasound holder to capture blood flow during dynamic RE, along with arterial diameter measurements before and immediately after dynamic RE. Lower limb RE elicited a prominent increase in anterograde shear rate but only modest increases in retrograde shear rate during exercise, followed by a transient hyperaemic response during recovery. These findings challenge our initial hypothesis, which predicted pronounced oscillatory flow velocity during exercise, and highlight predominantly anterograde blood flow despite rhythmic muscle contractions. As a secondary analysis, we tested for sex‐based differences and observed no differences in how females and males haemodynamically respond to RE. By measuring blood flow responses throughout dynamic RE, this study suggests that the vascular environment during RE closely resembles that of aerobic exercise, albeit with intermittent periods of reactive hyperaemia. This distinctive shear exposure pattern may partially contribute to the vascular changes observed with chronic RE training, although additional training models are needed to uncover the underlying mechanisms.

During muscular contractions, blood flow is characterized by a highly oscillatory, phasic pattern, resulting in fluctuating shear stress that can elicit intermittent periods of retrograde flow (Gonzales et al., 2008; Heffernan et al., 2013; Thomas et al., 2020; Walløe & Wesche, 1988). Current dogma suggests that a consistent retrograde shear stress stimulus is not beneficial in modulating endothelial function; however, when paired with anterograde shear in a transient oscillatory pattern, the corresponding vascular stimulus may result in vascular adaptation over time (Asakura & Karino, 1990; Green et al., 2025; Thomas et al., 2020). However, a large amount of previous work has used blood flow through inactive muscle beds to make these findings, often measuring haemodynamics in the brachial artery during leg cycling, kicking or isometric exercise. A novel aspect of our study lies in the characterization of blood flow in the active limb during contraction in an ecologically valid exercise setting. We captured continuous blood flow that revealed an oscillatory shear rate pattern during the contraction phases of RE, followed by a period of reactive hyperaemia during rest periods. The shear patterns we observed during RE (Figure 6) are similar to those previously reported during aerobic exercise (Thomas et al., 2020), contrary to our initial expectation of observing a more violent oscillatory pattern during leg press due to large swings in perfusion pressure. In fact, based on the flow stimulus alone, one would not expect drastic differences in the chronic vascular response to RE and aerobic exercise. Nevertheless, meta‐analyses point towards clear differences in the effect of exercise modality on vascular health, with RE having a smaller, or perhaps even negative, effect on large conduit artery function (Ashor et al., 2015; Miyachi, 2013). The variability in training‐related outcomes for a similar flow pattern stimulus underscores aspects of the haemodynamic environment that is not captured by conventional volume flow techniques. Additional vascular stimuli may be more relevant to describe the haemodynamic environment during exercise: for instance, turbulent blood flow generated during rhythmic muscle contraction, high transmural pressures that impart radial stress on endothelial cells, or the unique exercise and post‐exercise hormonal environment characterized by high inflammation compared to aerobic exercise (Green et al., 2017; Kojda & Hambrecht, 2005; Kraemer & Ratamess, 2005; Walløe & Wesche, 1988). Additional long‐term training studies are needed to clarify the contribution of these flow patterns to functional vascular responses following chronic exercise. By enabling stable assessment of local blood flow responses, a limb‐affixed probe holder enhances methodological precision in evaluating the mechanical–vascular interface and may help clarify how episodic retrograde shear and reactive hyperaemia shape endothelial signalling and long‐term vascular remodelling.

Novel to our findings, we detail the specific timing of blood flow changes across two common resistance training protocols: higher‐load lower‐repetition (75% 1‐RM for 8 reps) or lower‐load higher‐repetition (35% 1‐RM for 20 reps). While we initially theorized that the loading scheme might modify the blood flow pattern due to differences in extravascular pressure or set duration (Hanson et al., 2020), we did not observe differences in the blood flow response to the different protocols. Regardless of protocol, there was substantial oscillatory blood flow velocity behaviour during exercise that was likely modified by transient changes in downstream peripheral resistance (muscular contractions eliciting retrograde flow behaviour) and upstream changes in perfusion pressure (muscular contractions accentuating an arterial muscle pump); however, we did not explicitly measure upstream or downstream mechanisms in our study. From our data, it does not appear that the higher loading protocol accentuated the oscillatory response. Rather, we observed consistent exposure to oscillatory blood flow and transient post‐exercise hyperaemia resulting in short‐term increases in SFA diameter only during the rest periods, which was attenuated at the cessation of the exercise sets themselves. Over time, this exercise stimulus can result in chronic increases in resting vessel diameters and blood flow, which regress quickly with detraining (Stebbings et al., 2013). Taken together, our findings indicate that RE performed to failure produces a consistent oscillatory and hyperaemic stimulus, independent of loading scheme, which may represent a key haemodynamic signal underlying longer‐term vascular changes.

We did not observe any notable sex differences in the blood flow response to RE exercise, beyond the known larger arterial diameter in males. It is established that there are known sex‐based differences in resting vascular function, such as oestrogen‐mediated vasodilation, enhanced endothelial function and greater capillary density in females (Ansdell et al., 2020). Theoretically, females might have a greater propensity to increase blood flow during exercise, enhanced through oestrogen‐mediated vasodilation (Parker et al., 2007), though this effect was not replicated in a recent study using propranolol to block β‐adrenergic receptors in females during aerobic exercise (Doherty et al., 2025). In our study, the temporal patterns of vascular responses to RE were not different between sexes and independent of loading scheme.

### 4.1 Limitations

Participants were excluded if the leg press rack alone exceeded 35% of their 1‐RM, limiting our findings to individuals with prior leg press experience. Although the participants were required to keep their legs elevated throughout the entirety of the protocol to minimize movement and prevent displacement of the PFD device, this positioning represents a key limitation of our study. Elevating the legs above the heart has been shown to attenuate microvascular dilator responses and reduce exercise‐induced hyperaemia, potentially influencing the observed vascular results (Marume et al., 2023). We observed an increase in blood flow following exercise onset and transient increases in SFA diameter during rest periods, but it is possible that these effects may have been even more pronounced had the participants lowered their legs. In addition, while the PFD device is a valuable tool for capturing blood velocity during exercise and generally remains fixed in the desired position, device stability remains a concern for data interpretation. During certain resistance efforts it was observed that the device occasionally shifted as participants brought their legs back, disrupting the imaging plane. This issue was especially pronounced during higher efforts, where increased force and movement made maintaining consistent placement more difficult. Future studies should consider using a more controlled, isolated movement, such as a seated single‐leg press, to minimize excessive motion and reduce the likelihood of device displacement. Reduced limb motion would also improve arterial diameter imaging, which could not be measured in this study due to poor temporal resolution of the B‐mode imaging; as such, the interpretations of data taken during exercise sets should be taken with caution and primarily reflect velocity‐derived pattern changes rather than bulk blood flow changes. In addition, the location of lower‐limb arteries poses a challenge for device placement, as in pilot testing, the deep femoral artery exhibited too great a motion artifact to be reliably tracked during exercise, and the popliteal artery is positioned too close to the joint to allow full range of motion with probe holder use. Finally, the addition of an aerobic exercise condition would have allowed for direct comparison between modalities without inferring from previous reports.

## 5 Conclusion

While it is well established that exercise, in any form, provides significant cardiovascular benefits, each modality elicits distinct haemodynamic and vascular responses. For the first time, we present a full haemodynamic profile of two RE protocols, changing our perception of what RE induces within the active vasculature. It remains unclear whether the primary driver of vascular changes in response to RE training is the acute shear stress stimulus experienced during exercise, the cumulative shear load that persists in the post‐exercise recovery period, or other pressor or neurohormonal factors that change depending on the exercise modality. Future research should aim to characterize the diverse shear stress patterns induced by RE and investigate its long‐term vascular effects and adaptations. The growing popularity of RE, as well as its inclusion in public health guidelines (Ross et al., 2020), underscores the importance of understanding its unique cardiovascular implications.

## AUTHOR CONTRIBUTIONS

This study was completed in the VORTEX Lab (PI: Jason S. Au) at the University of Waterloo. Eudoxia Zafiris, Jeremy N. Cohen and Jason S. Au conceived and designed the study. All authors participated in material preparation, data collection, and interpretation of the data. Eudoxia Zafiris conducted all analyses. Jason S. Au was responsible for funding acquisition and supervision. The first draft of the manuscript was written by Eudoxia Zafiris and all authors provided critical feedback during drafting. All authors: read and approved the final manuscript; agree to be accountable for all aspects of the work in ensuring that questions related to the accuracy or integrity of any part of the work are appropriately investigated and resolved; and, agree that all persons designated as authors qualify for authorship, and all those who qualify for authorship are listed.

## CONFLICT OF INTEREST

The authors declare no financial conflicts of interest. The ProbeFix Dynamic was provided to the research team as an in‐kind contribution from Usono Inc. Usono Inc. did not have input on the design of the study or the reported results.

## GENERATIVE AI STATEMENT

No generative AI tools were used in the preparation of this article.

## Supporting information



Supplemental Video S1 is available at Figshare: https://doi.org/10.6084/m9.figshare.32826791

## Data Availability

Data are available for sharing upon request to the corresponding author.
