# Supplementary material for: Lower limb blood flow responses during higher‐ and lower‐load bilateral leg press exercise
Source: Exp Physiol. 2026 Aug 11:10.1113/EP092888. Online ahead of print. doi: 10.1113/EP092888 (PMC13458305; doi:10.1113/EP092888)
Supplement: Supplementary file 1 [file EPH-9999-0-s001.pdf]

Open Science Badges

Authors of articles accepted by Experimental Physiology are eligible to earn badges that recognize the authors' Open Research practices regarding the sharing of data or preregistered research plans. Please read more about the badges in our Information for Authors, and you can also find information on the Open Science Framework wiki.

Please check this box if you are interested in participating.

To apply for one or more badges acknowledging Open Research practices, please check the box(es) corresponding to the desired badge(s) below and provide the information on the availability and location of the data in the Data Availability Statement in your manuscript file. To qualify for an Open Data badge, you must provide a URL, DOI, or other permanent path for accessing the specified information in a public, open-access repository. Please read the Information for Authors to ensure the repository complies.

Authors who wish to publicly post third-party material in their data must have the proper authority or permission agreement in order to do so.

Preregistered Badge

1. Provide the URL, DOI, or other ***permanent path*** *for accessing the data in a* ***public, open-access repository****:*
DOI:10.17605/OSF.IO/VG82A

By signing below, authors affirm that the above information is accurate and complete, that any third-party material has been reproduced or otherwise made available only with the permission of the original author or copyright holder, and that publicly posted data do not contain information that would allow individuals to be identified without consent.

Jason Au

Date completed: 07/02/2026 13:18:01
User pid: 9788
